# Hyperoxia prevents the dynamic neonatal increases in lung mesenchymal cell diversity

**DOI:** 10.1038/s41598-023-50717-w

**Published:** 2024-01-23

**Authors:** Fabio Zanini, Xibing Che, Nina E. Suresh, Carsten Knutsen, Paula Klavina, Yike Xie, Racquel Domingo-Gonzalez, Min Liu, Alexander Kum, Robert C. Jones, Stephen R. Quake, Cristina M. Alvira, David N. Cornfield

**Affiliations:** 1 School of Clinical Medicine, University of New South Wales, Sydney, Australia; 2 Cellular Genomics Futures Institute, University of New South Wales, Sydney, NSW Australia; 3 Evolution & Ecology Research Centre, University of New South Wales, Sydney, NSW Australia; 4 Center for Excellence in Pulmonary Biology, Stanford University School of Medicine, Stanford, CA USA; 5 Division of Pulmonary, Asthma and Sleep Medicine, Department of Pediatrics, Stanford University School of Medicine, Stanford, CA USA; 6 Division of Critical Care Medicine, Department of Pediatrics, Stanford University School of Medicine, Stanford, CA USA; 7 School of Biochemistry and Immunology, Trinity College Dublin, Dublin, Ireland; 8 Department of Bioengineering, Stanford University, Stanford, CA USA; 9 Chan Zuckerberg Biohub, San Francisco, CA USA; 10 Department of Applied Physics, Stanford University, Stanford, CA USA

**Keywords:** Transcriptomics, Respiratory tract diseases, Physiology, Respiration

## Abstract

Rapid expansion of the pulmonary microvasculature through angiogenesis drives alveolarization, the final stage of lung development that occurs postnatally and dramatically increases lung gas-exchange surface area. Disruption of pulmonary angiogenesis induces long-term structural and physiologic lung abnormalities, including bronchopulmonary dysplasia, a disease characterized by compromised alveolarization. Although endothelial cells are primary determinants of pulmonary angiogenesis, mesenchymal cells (MC) play a critical and dual role in angiogenesis and alveolarization. Therefore, we performed single cell transcriptomics and in-situ imaging of the developing lung to profile mesenchymal cells during alveolarization and in the context of lung injury. Specific mesenchymal cell subtypes were present at birth with increasing diversity during alveolarization even while expressing a distinct transcriptomic profile from more mature correlates. Hyperoxia arrested the transcriptomic progression of the MC, revealed differential cell subtype vulnerability with pericytes and myofibroblasts most affected, altered cell to cell communication, and led to the emergence of *Acta1* expressing cells. These insights hold the promise of targeted treatment for neonatal lung disease, which remains a major cause of infant morbidity and mortality across the world.

## Introduction

Lung development requires coordinated interactions between multiple cell types in the epithelial, endothelial, immune and mesenchymal compartments^1,2^. In utero, the lung is fluid filled and the pulmonary circulation develops in a relatively low oxygen tension environment with low blood flow and high pressure^3^. Within moments after birth, the perinatal lung undergoes a remarkable transition that enables gas exchange with establishment of a gas–liquid interface, a tenfold increase in pulmonary blood flow and a marked decrease in pulmonary arterial pressure^4,5^. After birth, the distal lung undergoes structural remodeling including the formation of alveoli by secondary septation and a transition from a double to single capillary layer to increase gas exchange efficiency^6^.

Lung development requires coordinated interactions between multiple cell types in the epithelial, endothelial, immune and mesenchymal compartments^1,2^. Lung mesenchymal cells (MC) play a central role in secondary septation, thinning of the interstitium, and transmitting mechanical forces that promote alveolarization^7^. Although the lung mesenchyme includes multiple, distinct cell types, knowledge surrounding the degree of cellular heterogeneity, dynamic changes in gene expression, and cell–cell interactions during alveolarization remains limited.

Insight into the lung mesenchyme during early postnatal life has significant implications for lung injury and regeneration, across the lifespan, but especially in neonates. Since the lung continues to develop in late pregnancy and through the first decade of life^8^, premature infants are uniquely susceptible to life-threatening lung disorders, including bronchopulmonary dysplasia (BPD), a lung disease characterized by compromised alveolarization^9^. Given the importance of the mesenchyme in both physiologic development and developmental disease, we sought to interrogate the lung mesenchyme relative to cellular diversity, dynamic changes in gene expression and cell–cell communication in the perinatal lung, during alveolarization, and in the context of hyperoxia-induced lung injury, a preclinical model of BPD^10^.

In this report, we combined single cell transcriptomics (scRNA-Seq) with fluorescent multiplexed in situ hybridization (FISH) and computational analyses to characterize changes in composition, localization, and function of MC in the murine lung from just before birth through the first three weeks of postnatal life. Moreover, we applied the same strategies to cells derived from neonatal murine lung after 7 days of hyperoxia (80% oxygen). Consistent with prior studies, MC fell into three broad categories: fibroblasts, airway smooth muscle (ASM)/myofibroblasts (MyoF), and mural cells. Overall, the present strategy identified 14 distinct subtypes, some of which were restricted to perinatal developmental stages. Remarkably dynamic changes in cell phenotype occurred at birth as separate precursor cells for either fibroblasts or ASM/MyoF led to distinct but related cell subtypes between E18.5 and P1. At P7, early ASM cells were localized not only around the airways, but also in the distal lung, spatially proximate to, but transcriptionally distinct from, MyoF. At P7 lung MC were most proliferative and corresponded to the emergence of a novel proliferating population of MyoF. Hyperoxia blocked the maturation-related transcriptomic progression of MC, mirroring the structural arrest in lung development that characterizes BPD in infants, and markedly decreased pericyte and myofibroblast abundance and proliferation. Further, single-cell and bulk transcriptomics and FISH imaging identified previously undescribed *Acta1*+ MC in hyperoxia-exposed mice. These data demonstrate that the neonatal lung mesenchyme possesses a high degree of cellular diversity and changes dynamically with development, with hyperoxia causing both generalized transcriptomic arrest and the emergence of novel, previously undescribed MC.

## Results

### The perinatal lung mesenchyme is composed of three groups of cell types

**Figure 1:**
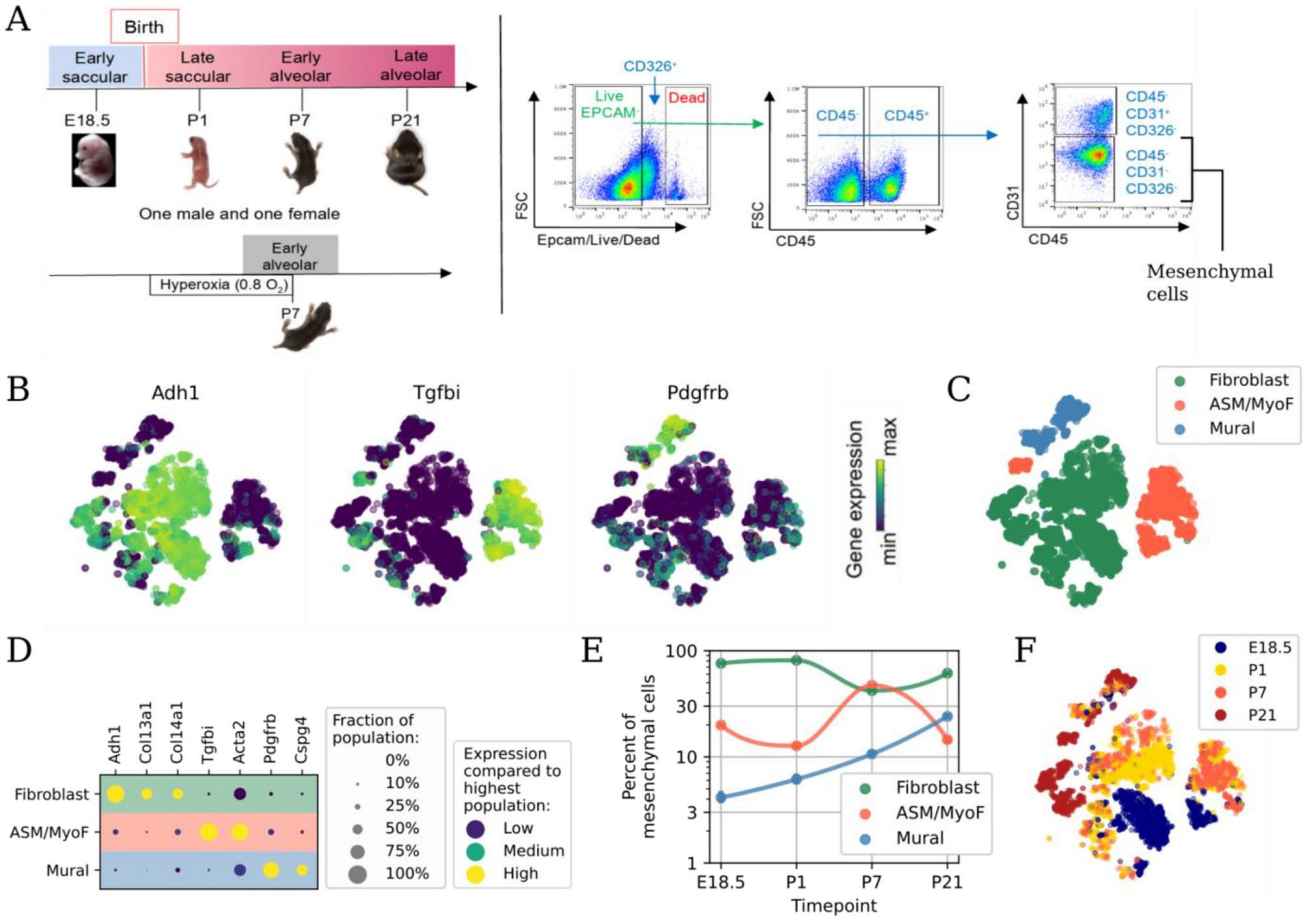
The perinatal lung mesenchyme is populated by three groups of cell types. (**A**) Experimental design including time points sampled, tissue dissociation, and cell sorting to isolate mesenchymal cells. (**B**) Embedding of lung mesenchymal cells, colored by relative expression of four distinguishing marker genes. (**C**) As in (**B**), but colored by cell type group. (**D**) Dot plot of representative marker genes for each group. (**E**) Relative abundance for each group in normal development. (**F**) As in (**B**), but colored by time point of each cell. At this level of resolution, airway smooth muscle (ASM) and myofibroblasts (MyoF) belong to the same group (ASM/MyoF).

Single cell RNA-Seq (scRNA-Seq) data were generated from eight mice at different stages of perinatal development, with two mice (one female and one male) from four points during development, embryonic day 18.5 (E18.5; early saccular stage), postnatal day of life 1 (P1; late saccular), P7 (early alveolar) and P21 (late alveolar). Lung tissue was resected, perfused, and dissociated as described previously^11^. Fluorescence activated cell sorting (FACS) was used to enrich mesenchymal cells by depleting cells positive for CD326, CD31, and CD45 (Fig. 1A). Libraries were generated in 384-well plates, combining medium cell throughput with excellent per-cell capture sensitivity as demonstrated previously^11^. A total of 5493 high-quality cells at an average depth of 980,000 read pairs per cell were analyzed (see “Materials and methods” section), yielding a much larger number of detected genes per cell compared to any other study of the perinatal lung (Fig. S1). Key reagents used in the study are included in Table 1.

**Table 1:** Reagents.

Reagent type (species) or resource	Designation	Source or reference	Identifiers	Additional information
Strain, strain background (M. musculus)	C57BL/6J	The Jackson Laboratory	Stock No:000664	
Commercial assay or kit	Liberase TM TL Research Grade	Sigma Aldrich	Cat No. 5401020001	
Antibody cocktail	Purified Anti-Mouse CD16/CD32 (Fc Shield; Rat monoclonal)	Tonbo Biosciences	Clone: 2.4G2 Cat No. 70-0161-U100	Pre-FACS (1:100)
Antibody	Anti-CD31 (Rat monoclonal)	BioLegend	Clone: MEC13.3 Cat No. 102501	FACS (1:100)
Antibody	Anti-CD326 Monoclonal Antibody, eFluor 450	eBioscience/ThermoFisher	Clone: G8.8 Cat# 48-5791-82	FACS (1:100)
Antibody	Anti-CD45 (Rat monoclonal)	eBioscience/ThermoFisher	Clone: 30-F11 Cat#14-0451-82	FACS (1:100)
Commercial assay or kit	RNAscope® Multiplex Fluorescent Detection Kit v2	Advanced cell diagnostics (ACD)	Cat No. 323110	
Commercial assay or kit	Probe-Mm-Tgfbi	ACD	Cat No. 494551	RNAscope: 50 μl per slide
Commercial assay or kit	Probe-Mm-Pdgfra-C2	ACD	Cat No. 480661-C2	RNAscope: (1:50)
Commercial assay or kit	Probe-Mm-Hhip-C3	ACD	Cat No. 448441-C3	RNAscope: (1:50)
Commercial assay or kit	Probe-Mm-Crh-C2	ACD	Cat No. 418011-C2	RNAscope: (1:50)
Commercial assay or kit	Probe-Mm-Mki67-C3	ACD	Cat No. 416771-C3	RNAscope: (1:50)
Commercial assay or kit	Probe-Mm-Wnt2	ACD	Cat No. 313601	RNAscope: 50 μl per slide
Commercial assay or kit	Probe-Mm-Tubb3-C2	ACD	Cat No. 423391-C2	RNAscope: (1:50)
Commercial assay or kit	Probe-Mm-Cspg4-C3	ACD	Cat No. 404131-C3	RNAscope: (1:50)
Commercial assay or kit	Probe-Mm-Acta1-C3	ACD	Cat No. 808831-C3	RNAscope: (1:50)

An initial representation of the perinatal lung mesenchyme was constructed by computing a t-distributed stochastic neighbor embedding (t-SNE)^12^ and categorizing each cell based on expression of established marker genes (Fig. 1B). Three groups of mesenchymal cell types were defined (Fig. 1C). Fibroblasts were marked by *Adh1*^13,14^. *Tgfbi* expression marked a heterogeneous group of cells that included including both airway smooth muscle cells and myofibroblasts (ASM/MyoF)^15^. Mural cells were marked by *Pdgfrb*^16^ (Fig. 1D). At all time-points, except for P7, most lung mesenchymal cells were fibroblasts. Airway smooth muscle cells and myofibroblasts abundance peaked at P7, with mural cell abundance increasing slowly with development (Fig. 1E). Both cell type abundance and gene expression changed dynamically with maturation (Fig. 1F). No cluster coexpressed both *Tgfbi* and *Pdgfrb* (Fig. 1D), previously described as vascular smooth muscle cells (VSM) markers^17^, while airway smooth muscle cells and myofibroblasts embedded in distinct locations across development (Fig. 1F), underscoring the importance of smooth muscle cell subtype identification^18–21^.

### Perinatal and adult mesenchymal cell subtypes are transcriptionally distinct

**Figure 2:**
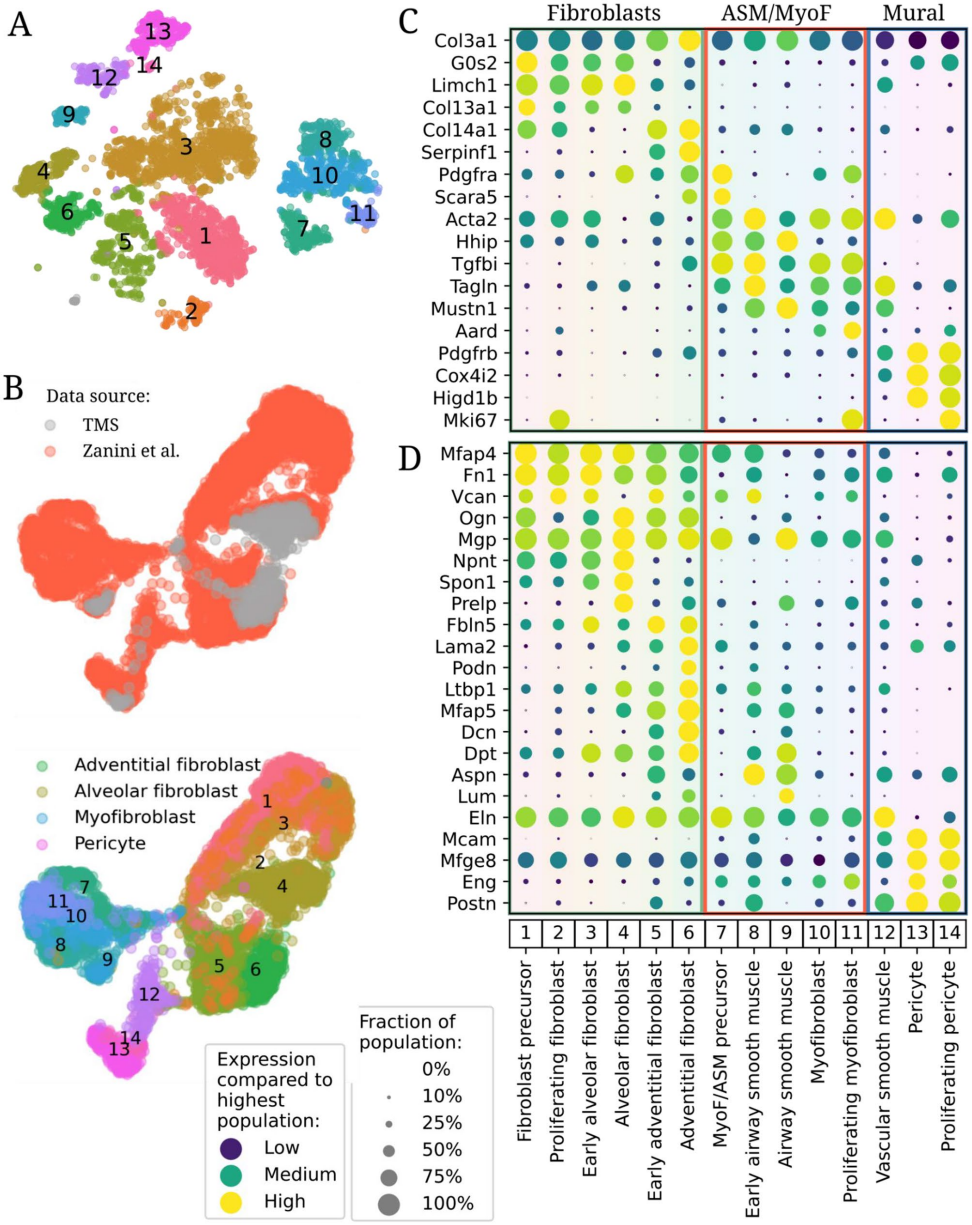
Harmonized fine grained composition of the perinatal mesenchyme. (**A**) Embedding as in Fig. 1B, but colored by fine cell type and annotated with cluster numbers 1–14. This color code is used in subsequent figures. (**B**) Harmonized embedding of perinatal mesenchymal cells with mesenchymal cells from TMS colored by source (top panel, gray TMS cells drawn on top) and cell type (bottom, merging concordant cell types between our data and TMS). (**C,D**) Dotplots of all mesenchymal cell types in this study for (**C**) marker genes and (**D**) components of the extracellular matrix. *TMS* Tabula Muris Senis.

We then undertook more discrete cell subtype annotation^18,19,21–23^. Leiden clustering^24^ resulted in 14 distinct clusters (Fig. 2A). Annotation was achieved by harmonized co-embedding with an adult cell atlas, Tabula Muris Senis (TMS, Fig. 2B)^19^ and marker gene analysis (Fig. 2C). Cell clusters at P21 cell were consistent with the adult atlas, while clusters derived from earlier time points (clusters 1–3, 5, 7–8, 10–11) embedded separately^25^, supporting the notion that perinatal lung cells possess transcriptomic profiles distinct from adult. Clusters 1–6 were consistent with fibroblasts, clusters 7–11 included airway smooth muscle (ASM) and myofibroblasts, and clusters 12–14 mural cells.

For cluster 12, which co-embedded between smooth muscle-like cells and pericytes (Fig. 2B), the coexpression of both smooth muscle (*Tagln*, *Acta2*) and mural (*Prgfrb*) genes (Fig. 2C) identified this population as vascular smooth muscle^26,27^. Cluster 9 derived from P21 mice (Fig. 1E) and expressed *Hhip*, an established airway smooth muscle marker^28^ but not *Pdgfra*, a myofibroblast marker, therefore these cells were annotated as ASM (Fig. 2C). This led to the tentative annotation of cluster 8 (*Hhip*+ *Pdgfra*−) as early ASM and cluster 10 (*Hhip*−* Pdgfra*+) as myofibroblasts (Fig. 2C). Both clusters comprised mostly cells from P1 and P7. At E18.5, a single *Tgfbi*+ cluster was found (cluster 7). It coexpressed *Hhip* and *Pdgfra* and was therefore annotated as ASM/MyoF precursors. Clusters 2, 11, and 14 expressed *Mki67* and were therefore labeled as proliferating fibroblasts, myofibroblasts, and pericytes, respectively (Fig. 2C). To contextualize our cell type definitions, we computed dot plots of marker genes from recent publications against our cell types^22,29^ (Figs. S2, S3).

To further buttress the physiologic relevance of the transcriptomic findings, expression of genes associated with extracellular matrix (ECM) production and remodeling were evaluated (Fig. 2D). Fibroblasts shared ECM components *Mfap4*, *Fn1, Vcan,* and *Ogn*. In alveolar fibroblasts (AlvF), subtype-specific expression included *Spon1* which encodes a secreted adhesion protein^30^. Mature and Early Adventitial fibroblasts (AdvF) expressed *Mfap5*, which contributes to tissue elasticity^31^, and *Dcn*, which confers resistance to tissue compression, while only adventitial fibroblasts from P21 mice expressed *Podn*, encoding a molecule that constrains smooth muscle cell proliferation and migration^32^. Early ASM highly expressed *Aspn*, which inhibits canonical TGFβ and SMAD signaling^33^ and competes with *Dcn* for collagen binding^34^, while ASM from P21 animals had reduced expression. However, mature ASM expressed *Lum*, which regulates collagen fibril organization. Interestingly, Early ASM, found in P1 and P7 mice, did not express *Lum*, suggesting other genes might regulate collagen fibrils at those time points^35,36^. Pericytes, which modulate vascular tone, endothelial cell (EC) migration and angiogenesis^37^, expressed *Mcam*, *Mfge8*, *Postn*, and *Eng*. Vascular smooth muscle had the highest expression of elastin (*Eln*), while all other cell types except pericytes expressed some level of *Eln*.

### Fibroblast and ASM/MyoF precursors differentiate at the onset of air-breathing life

**Figure 3:**
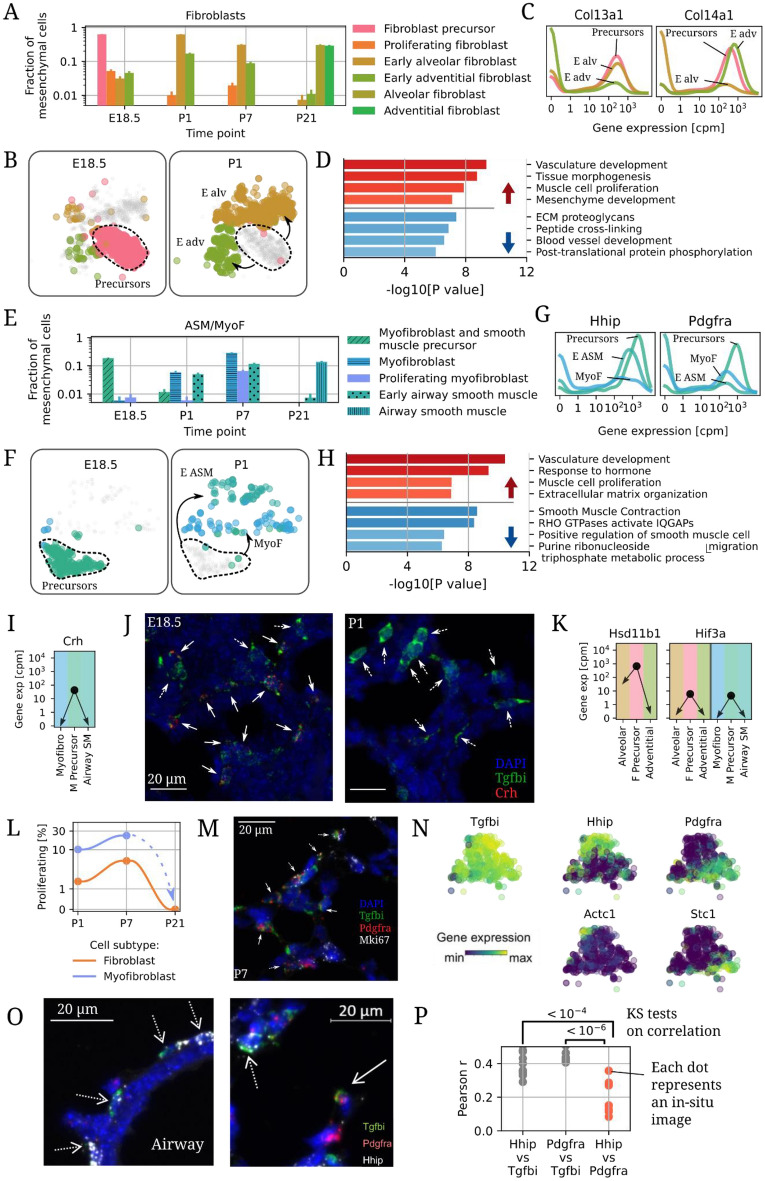
Developmental changes in perinatal fibroblasts, myofibroblasts, and airway smooth muscle. (**A**) Changes in composition of lung fibroblast subtypes between E18.5 and P21. (**B**) Embedding of fibroblasts and precursors at E18.5 (left) and P1 (right). (**C**) Distribution of expression for *Col13a1* and *Col14a1* in fibroblasts. (**D**) Pathways enriched among genes up- (red, top) or down-regulated (blue, bottom) in early postnatal fibroblasts versus prenatal fibroblast precursors^38^. (**E**) Changes in abundance of ASM, myofibroblasts and their precursors between E18.5 and P1. (**F**) Embedding of ASM and myofibroblasts at E18.5 (left) and P1 (right). (**G**) Distribution of expression of *Hhip* and *Pdgfra* in myofibroblasts, and early ASM, and their precursors. (**H**) Pathways enriched among genes up- (red, top) or down-regulated (blue, bottom) in early postnatal myofibroblasts and ASM versus their precursors. (**I**) Expression changes of *Crh* between E18.5 and P1. (**J**) In-situ hybridization of *Crh*+ *Tgfbi*+ (solid arrows) and *Crh- Tgfbi*+ cells (dashed arrows) in E18.5 and P1 lungs. (**K**) Expression changes of Hsd11b1 in fibroblasts and Hif3a in both fibroblasts, ASM, and myofibroblasts between E18.5 and P1. (**L**) Fraction of proliferative fibroblasts and myofibroblasts at postnatal time points. (**M**) In-situ hybridization of nonproliferating (*Mki67*− solid arrows) and proliferating (*Mki67*+ dashed arrows) myofibroblasts at P7. (**N**) Embedding of Early ASM and myofibroblasts, colored by subtype and by expression of select genes. (**O**) In-situ hybridization of P7 lungs around airways (left) and in the distal lung (right). Dashed arrows: *Tgfbi*+ *Hhip*+ cells, solid arrow: *Tgfbi*+ *Pdgfra*+ cells. (**P**) Quantification of the degree of coexpression of *Hhip*, *Tgfbi*, and *Pdgfra* from in-situ images of P7 mice. P-values are Kolmogorov–Smirnov tests with Bonferroni multiple hypothesis correction.

The cell composition of the lung changed dramatically between E18.5 and P1, a time window of just 48 h. Fibroblast precursors, the most abundant mesenchymal type at E18.5, disappeared by P1 (Fig. 3A), concomitant with the appearance of two distinct types of postnatal fibroblasts (Fig. 3B, Fig. S3) distinguished by expression of either *Col13a1* or *Col14a1* (Fig. 3C), resembling adult alveolar and adventitial fibroblasts, respectively. *Col13a1* and *Col14a1* were coexpressed in the E18.5 precursors, suggesting a common origin (Fig. 3C), a hypothesis corroborated by trajectory analysis via diffusion maps, pseudotime, and RNA velocity (Fig. S4). In terms of ECM-associated gene expression, fibroblast precursors appeared slightly more similar to alveolar fibroblasts (Fig. 2D, Fig. S4). Differentially expressed genes between embryonic and a balanced mix of postnatal fibroblasts showed a statistical enrichment of mesenchyme-, ECM-, and vascular development-associated pathways in both groups (Fig. 3D, red/blue bars upregulated in precursors/postnatal cells, respectively)^38^.

Airway smooth muscle/myofibroblast precursor abundance decreased between E18.5 and P1 (Fig. 3E). Early airway smooth muscle cells and myofibroblasts were rare before birth and increased in abundance by P1 (Fig. 3E,F). Airway smooth muscle/myofibroblast precursors coexpressed *Hhip* and *Pdgfra*, while postnatal MyoF expressed only *Pdgfra* and postnatal Early ASM expressed only *Hhip* (Fig. 3G). Pathway analysis suggested that ASM/MyoF precursors expressed higher levels of genes related to vascular development and muscle cell proliferation (Fig. 3H, red bars) and lower levels of smooth-muscle contraction genes (blue bars) compared to balanced postnatal ASM/MyoF cells. Airway smooth muscle/myofibroblast precursors but not postnatal counterparts expressed corticotropin releasing hormone (*Crh*, Fig. 3I), an early signal for glucocorticoid production within the hypothalamic–pituitary–adrenal (HPA) axis. *Crh* plays an essential role in lung maturation^39^ via cortisol-induced increases in surfactant protein release^40,41^. *Crhr1* and *Crhr2*, which encode the *Crh* receptors, were not expressed by any other cell type in the perinatal lung (Fig. S5)^11,17,42^, not in the adult atlas^19^. *Tgfbi*+ *Crh*+ cells were detected by in-situ hybridization imaging (RNA-Scope) within the lung parenchyma in E18.5 mouse embryos but not postnatally (Fig. 3J, solid arrows, n = 6 animals per group). Fibroblast precursors but not postnatal fibroblasts expressed high levels of *Hsd11b1* (Fig. 3K), the main enzyme responsible for converting cortisone into its bioactive form cortisol, indicating a local amplification of glucocorticoid action^43^. Expression of *Hif3a*, a negative regulator of *Hif1a*, was lost in both fibroblasts and ASM/MyoF cells after birth, suggesting tight regulation of this pathway at the critical transition between fetal development and air-breathing life (Fig. 3K).

*Mki67*+ proliferating myofibroblasts and pericytes were observed at P1 and increased in abundance at P7 (Fig. 3L). Proliferating myofibroblasts were localized throughout the parenchyma of P7 lungs as *Tgfbi*+ *Pdgfra*+ *Mki67*+ cells (Fig. 3M, dashed arrows, n = 2) adjacent to *Tgfbi*+ *Pdgfra*+ *Mki67*− nonproliferating myofibroblasts (solid arrows). There were no proliferating (myo-)fibroblasts detected via scRNA-Seq from P21 mice (Fig. 3L), consistent with a progression towards cell quiescence towards the end of alveolarization and adulthood.

The relationship between postnatal Early ASM and myofibroblasts was then examined in detail. These cells, in addition to shared *Tgfbi* expression and exclusive expression of *Hhip* (for Early ASM) and *Pdgfra* (for myofibroblasts) as mentioned above, showed partial expression of *Actc1*, an alpha actin typically expressed in heart muscle^44^, in a fraction of Early ASM and *Stc1*, a regulator of calcium levels that has been implicated in myopathy^45^, in a fraction of myofibroblasts, indicating further layers of latent transcriptomic heterogeneity (Fig. 3N). In-situ hybridization was then used to determine the location of each cluster at P7. As expected, only *Tgfbi*+ *Hhip*+ Pdgfra− cells, a profile matching Early ASM, were found encircling airways (Fig. 3O left, dashed arrows, n = 2), buttressing the scRNA-Seq-based annotation. Surprisingly, however, not only *Tgfbi*+ *Hhip*− *Pdgfra*+ (solid arrows), matching myofibroblasts, but also *Tfgbi*+ *Hhip*+ *Pdgfra*− cells (dashed arrows), matching Early ASM, were observed in the distal parenchyma (Fig. 3O, right, n = 2). Automated image analysis on the distal parenchyma only (10 images, no airways) demonstrated a lower correlation between *Pdgfra* and *Hhip* than the correlation of either molecule with *Tgfbi* (Fig. 3P), which mirrors the transcriptomics results (Fig. 3N).

### Mural cells change gradually during perinatal development

**Figure 4:**
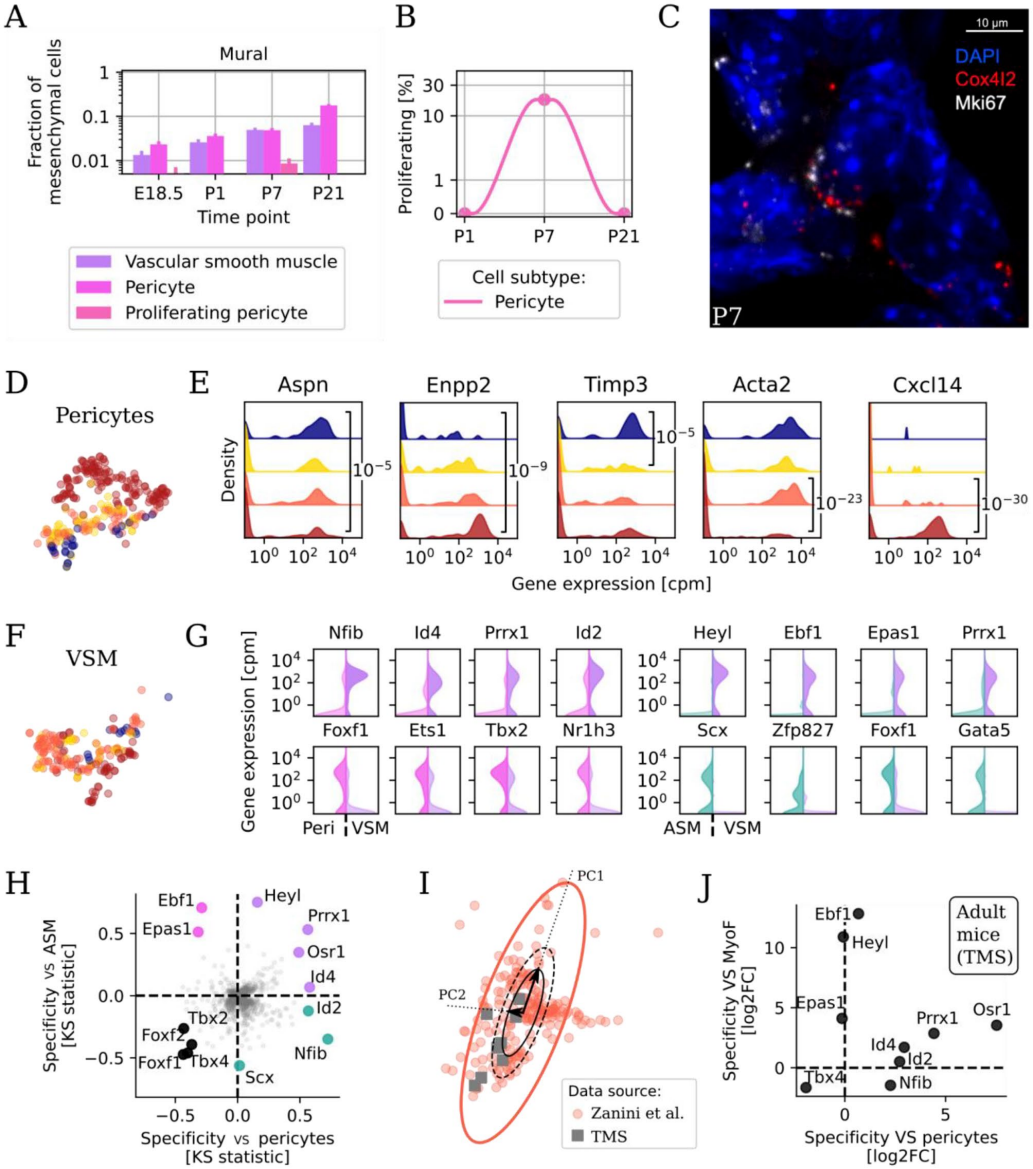
Mural cells encompass proliferating pericytes at P7 and harmonized vascular smooth muscle characterized by specific transcription factors. (**A**) Mural cell type composition between E18.5 and P21. Colors as in Fig. 2A. (**B**) Fraction of proliferating pericytes at postnatal time points. (**C**) Representative FISH image of *Mki67*+ *Cox4i2*+ proliferating pericytes in P7 lungs. (**D**) Embedding of pericytes, colored by time point. (**D**) Violin plots of the expression of select genes that change in expression over perinatal development in pericytes. (**F**) Embedding of VSM, colored by time point. (**G**) Violin plots of gene expression in pericytes versus VSM (left) and ASM versus VSM (right) for transcription factors (TFs) marking either cell type. (**H**) Combined statistical enrichment of expressed transcription factors in VSM versus pericytes (x axis) and versus ASM (y axis). Genes in the upper right quadrant (violet) are specific to VSM versus both ASM and pericytes, while genes in the lower left quadrant (black) are specifically absent in VSM but not in pericytes nor ASM. (**I**) Harmonized embedding of VSM from this study (red circles) and overlapping cells from Tabula Muris Senis (gray squares), identified using a rigidly expanded principle component analysis (PCA) ellipse. These cells are originally annotated as pericytes, myofibroblasts, or fibroblasts. (**J**) Differential expression of select transcription factors in newly identified atlas VSM versus atlas pericytes (x axis) and versus atlas myofibroblasts (y axis).

Mural cells, which include pericytes and vascular smooth muscle, form the outer lining of the vasculature and help support the endothelium and regulate vascular tone. In the perinatal lung, the transcriptomes of mural cells changed slowly between E18.5 and P21. Relative cell type abundance of mural cells (Fig. 1D) and of each mural subtype (pericytes and VSM) (Fig. 4A) increased steadily over time. A cluster of proliferating pericytes peaked in abundance at P7 (Fig. 4B), at the same time as proliferating myofibroblasts. Confocal imaging of P7 lung confirmed the presence of proliferating pericytes that co-expressed *Cox4i2* and *Mki67* (Fig. 4C, n = 2). A developmental shift in the pericyte transcriptome was visible in our embedding (Fig. 4D, colors as in Fig. 1F). Further analyses revealed specific transcriptomic progressions: genes exhibiting gradual decreases in expression over time (e.g. *Aspn*), gradual increases over time (e.g. *Enpp2*), marked downregulation after birth (e.g. *Timp3*), and either marked down-regulation (e.g. *Acta2*) or up-regulation (e.g. *Cxcl14*) between P7 and P21 (Fig. 4E, same colors—all P-values are Bonferroni-adjusted KS tests). In contrast, VSM cells did not exhibit a clear transcriptomic shift over time in the embedding (Fig. 4F) and lacked high expression of *Mki67* (Fig. 2A).

The transcriptional distinction between VSM, pericytes, and ASM remains incompletely defined^18,19,26,29^. For instance, Guo et al. annotated as “Pericyte-1” a cell cluster which lacks pericyte markers (Fig. S6). To clarify the transcriptional identity of VSM, our analysis focused on transcription factors (TFs) given the capacity to effectively distinguish between cell types^20^. Several transcription factors were upregulated in VSM (purple) relative to ASM (green) and in VSM compared to pericytes (pink) (Fig. 4G, top) or downregulated in VSM versus the same cell types (Fig. 4G, bottom). VSM also expressed TFs that were absent in both ASM and pericytes (Fig. 4H, purple dots): these included *Prrx1*^46^, *Osr1*^47^ and *Id4*^48^. *Tbx4*^49^ and other genes were expressed in both ASM and pericytes but specifically downregulated in VSM (black dots). Given the absence of a VSM annotation in adult atlas^19^, we reanalyzed atlas cells that overlapped in the harmonized embedding (Fig. 2B) with our VSM cells (Fig. 4I). The 8 atlas cells within the principal component analysis-based ellipse (gray squares) were originally annotated as adult myofibroblast or pericyte. However, the co-embedding coordinates overlapped with those of our VSM (red circles). Differential expression of transcription factors between these 8 atlas cells and other atlas pericytes and myofibroblasts resulted in a specific TF fingerprint (Fig. 4J) that was almost identical to the developmental VSM fingerprint previously defined (Fig. 4H), with upregulation of *Prrx1*, *Osr1* and *Id4*, and downregulation of *Tbx4* compared to both other cell types. In-situ hybridization of P7 lungs with *Tgfbi*, *Pdgfra*, and hydrazide (which marks vessels but not airways) showed that cells lining the large airways highly expressed *Tgfbi* (which is expressed by both ASM and myofibroblasts), but cells located between the double elastic laminae of arterioles were *Tgfbi* negative (Fig. 4, Supplementary Fig. 2). Overall, these data confirmed that VSM differ from both ASM and myofibroblasts not only in their location within the tissue but also in their transcriptional profile, in agreement with some reports^26,50^.

### Hyperoxia inhibits mesenchymal transcriptional progression and pericyte proliferation

**Figure 5:**
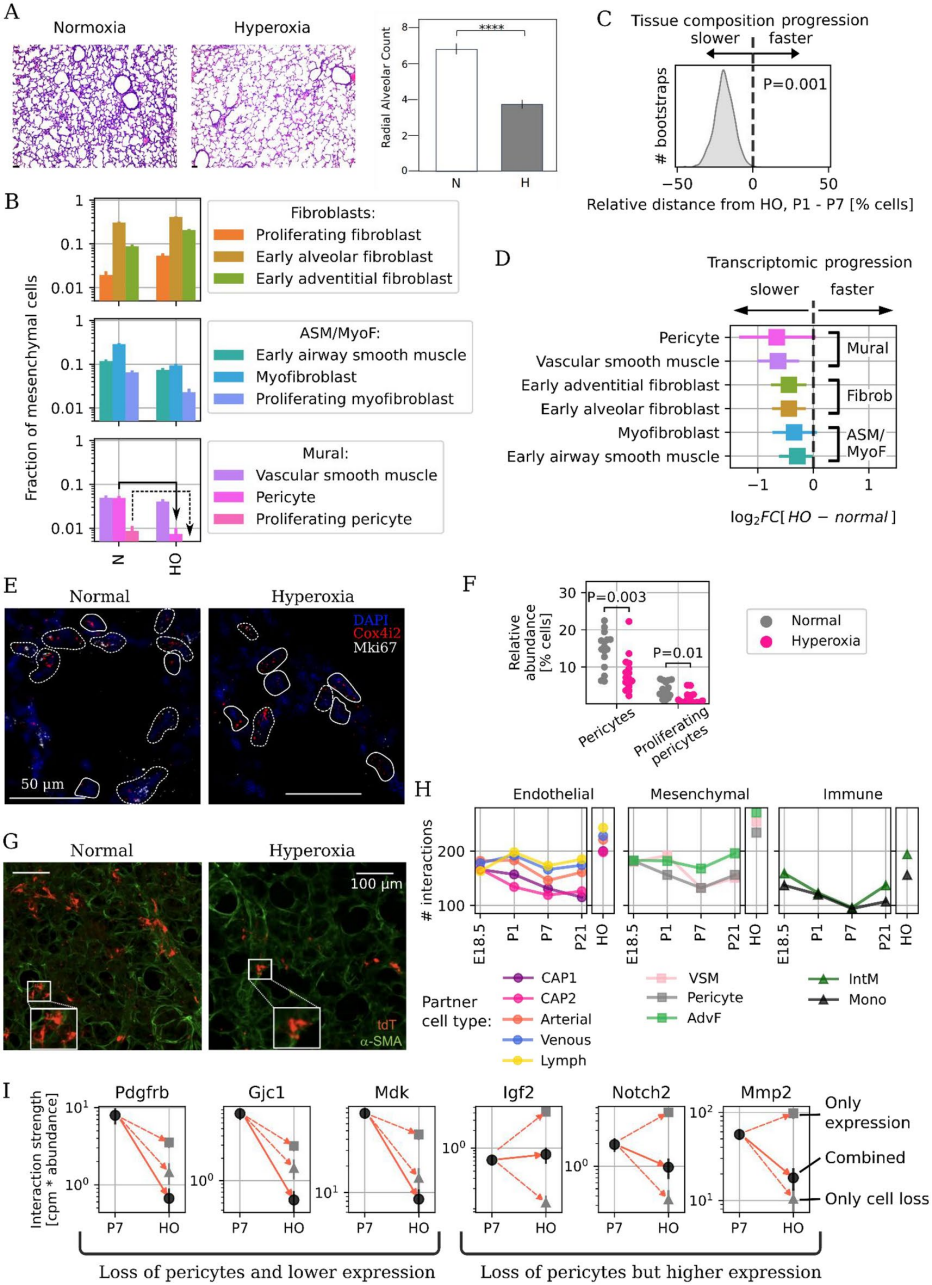
Hyperoxia delays mesenchymal transcriptional progression and suppresses pericyte proliferation. (**A**) Representative histologic sections from murine lungs developing in normoxia (left) or hyperoxia (right) through P7 and radial alveolar counts in normoxic (N) and hyperoxic (HO) P7 mouse lungs. Scale bar: 50 μm. ****P-value < 0.001. (**B**) Cell subtype composition in between normoxia and hyperoxia at P7. (**C**) Cell subtype composition changes in hyperoxic mice. The arrows indicate the observed severe loss of pericytes (solid) and complete loss of proliferating pericytes (dashed). (**D**) Kernel density estimate of 1000 bootstraps over cells (balanced at each time point) for the difference in distance on cell composition between hyperoxic mice and P7, minus hyperoxic mice and P1. A positive number indicated that the hyperoxic mesenchyme composition is closer to a healthy P1 mouse than a P7 one. Only 1 out of 1000 simulations showed hyperoxia closer to P7 than P1. (**D**) Differential expression between normoxic and hyperoxic cells in each cell type for genes progressing during early postnatal development (upregulated at P7 versus P1). (**E**) Representative in-situ images of pericytes (solid) and proliferating pericytes (dashed) in P7 normoxia and hyperoxia mice. (**F**) Quantification of the relative abundance of pericytes and proliferating pericytes in in-situ images from P7 mice using an unsupervised image analysis approach over thousands of cells. (**G**) Representative confocal imaging of lung tissue at P7 from Notch3-CreERT2-tdT expressing mouse pups who received tamoxifen from P1 to P3 and were placed in either normoxia or hyperoxia for seven days from birth to P7. ⍺-smooth muscle actin (⍺-SMA) shown in green. (**H**) Number of detected ligand-receptor cell–cell interactions between pericytes and other cell types at each time point during normal development and in hyperoxia mice at P7. (**I**) Strength of select interactions with pericytes measured as product of gene expression and pericyte abundance in normoxia and hyperoxia. Data sources: endothelial cells^17^ and immune cells^11^.

To study the discrete transcriptomic effect of hyperoxia on the developing lung, we performed scRNA-Seq and in-situ analysis on mice exposed to 80% oxygen (hyperoxia, HO) from birth through P7^10,51–53^. Early lung growth was impaired by hyperoxia with larger and fewer alveoli in mice exposed to HO, compared to normoxia, while the radial alveolar count was lower (Fig. 5A; P < 0.001, n = 3, student’s test) in hyperoxic compared to normoxic mice. Hyperoxia increased the relative abundance of fibroblasts but decreased the abundance of ASM, proliferating myofibroblasts and pericytes (Fig. 5B). We hypothesized that hyperoxia might render the lung cellular composition at P7 more similar to younger animals. We compared cell type abundance profiles via bootstrapping and found that the transcriptomes of hyperoxia-exposed mice were significantly more similar to healthy P1 mice than P7 mice (P = 0.001, Fig. 5C), indicating that hyperoxia inhibited the cellular diversification of the mesenchyme. We next asked whether hyperoxia delayed developmental changes in the transcriptome within each cell type. We specifically interrogated the differential expression of genes that exhibit transcriptional progression during development in normoxia (e.g. higher at P7 than P1), and found that hyperoxia inhibited this progression in all the mesenchymal populations, especially in the mural cells (Fig. 5D).

Hyperoxia dramatically decreased pericyte number and especially proliferating pericytes. The scRNA-Seq data (Fig. 5B, solid and dashed arrows, respectively) were confirmed in vivo (Fig. 5E, n = 2 for each group). Hyperoxia reduced the abundance of both total pericytes (P = 0.003, KS test) and proliferating pericytes (P = 0.01, KS test) (Fig. 5F, each dot represents an analyzed image). To further confirm this observation Notch3-CreERT2-tdT mice were given tamoxifen at P0 and P3 and placed in normoxia or hyperoxia from until P7. Deep imaging of thick distal lung section (150 μm) indicated that compared to normoxia, pericyte number was markedly reduced in hyperoxia (Fig. 5G, representative images of n = 3 animals for each group), and the morphology of the pericytes disrupted, with a decrease in the number and length of projections (Fig. 5G, high mag inset).

To determine whether pericyte loss might affect the cell–cell communication networks, we used the CellPhoneDB database^54^ to count the number of putative ligand–receptor interactions (at least 20% of cells expressing each gene) between pericytes and EC, other mesenchymal cells, and immune cells at all four time points and in hyperoxia at P7. Hyperoxia increased the number of putative interactions between remaining pericytes and all other cell types (Fig. 5H). The overall “interaction strength”, the product of cell type abundance and gene expression levels, varied by gene (Fig. 5I). For some genes, down-regulation intensified the effect of pericyte loss, possibly leading to loss of signaling. This analysis demonstrated a potential decrease in signaling for *Pdgfrb*, an essential component of a pathway promoting pericyte proliferation and migration towards the endothelium during vascular development^55^, *Gjc1*, a connexin that promotes vessel stability and EC quiescence^56^, and midkine or *Mdk*, a heparin binding, proangiogenic cytokine^57^. In contrast, other genes exhibited up-regulation of gene expression, which may serve to mitigate the effect of diminished pericyte type abundance and thereby preserve signaling.

### Hyperoxia can cause the emergence of *Acta1*+ cells in the neonatal lung

**Figure 6:**
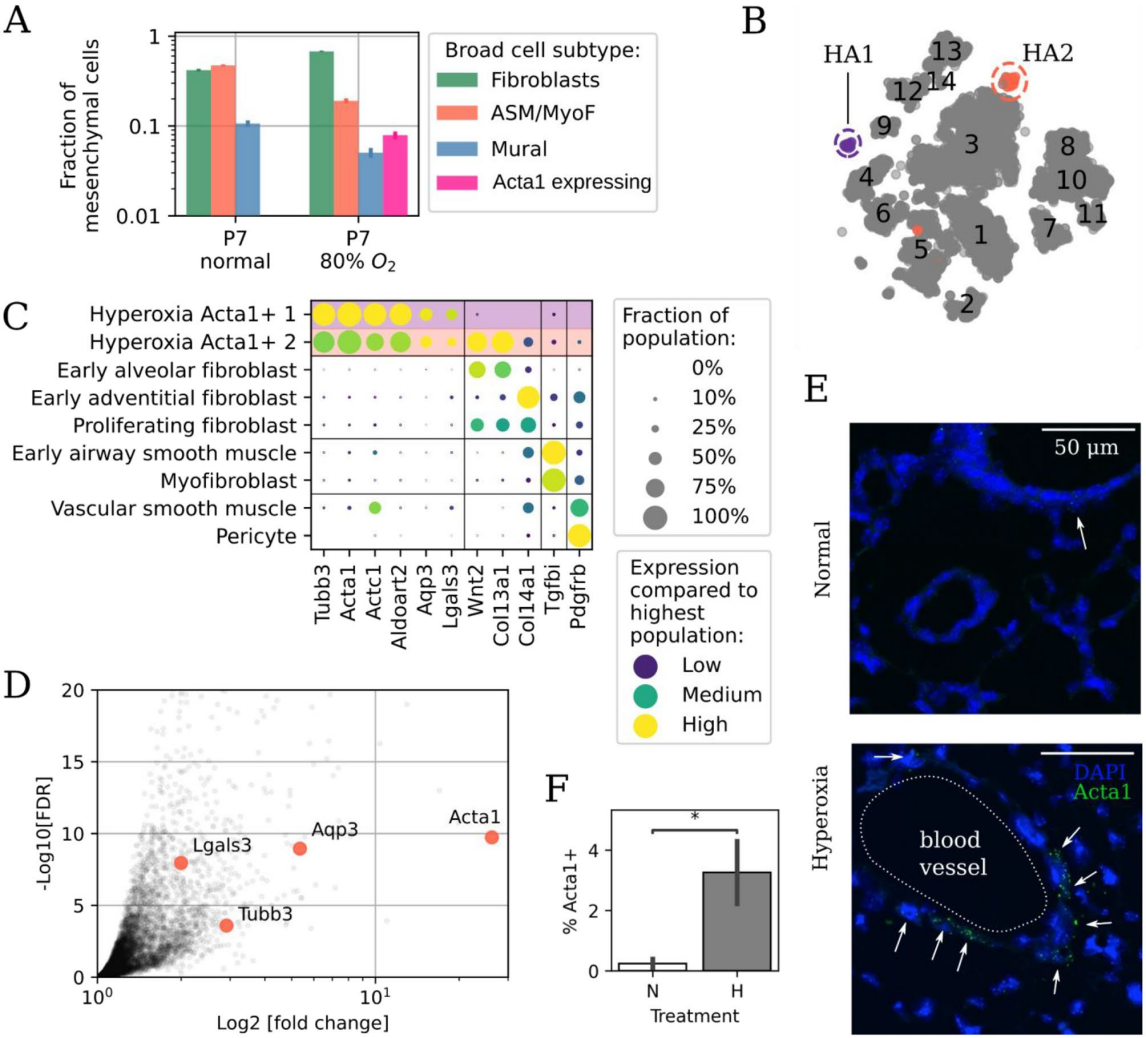
Hyperoxia causes the emergence of *Acta1*+ cells in the lung. (**A**) Relative cell subtype abundance for broad mesenchymal types in normoxia and hyperoxia. (**B**) Embedding as in Fig. 2A but with the additional populations hyperoxic *Acta1*+ 1 (HA1, in purple) and hyperoxic *Acta1*+ 2 (HA2, in red). Other cells are colored in gray. (**C**) Dot plot of marker genes for *Acta1*+ cells. (**D**) Reanalysis of bulk transcriptomics in P7 lungs exposed to hyperoxia shows up-regulation of *Acta1* and other marker genes for the novel cell populations. Data from Ref.^59^. *FDR* false discovery rate. Downregulated genes are not shown. (**E**) Representative in-situ hybridization images of *Acta1* (green) expression in normoxia and hyperoxia. Cells with three or more *Acta1* molecules are indicated by arrows. (**F**) Quantification of percentage of *Acta1*+ cells in images from normoxic and hyperoxic mice. *P-value < 0.05.

In hyperoxic mice, novel populations of Acta1+ cells were observed (Fig. 6A). The Acta1+ cells have not been previously described during lung development or in hyperoxia^18^. The fraction of mesenchymal cells expressing Acta1+ cells is significantly greater in hyperoxia, compared to normoxia (Fig. 6A; P < 0.001). The two novel clusters (henceforth called HA1 and HA2) were projected in distinct parts of the embedding, with HA2 located close to Alveolar fibroblasts (Fig. 6B). Both clusters expressed the neuron-specific tubulin (*Tubb3*) and *Lgals3*, encoding a -galactosidase binding lectin that is up-regulated in tissue fibrosis^58^ as well as the cardiac muscle gene *Actc1* and the membrane channel *Aqp3*. The HA2 cluster also expressed genes characteristic of alveolar fibroblasts (e.g. *Wnt2*, *Col13a1*) (Fig. 6C), explaining its embedding proximity to fibroblasts. *Acta1*+ cells did not express endothelial (e.g. *Pecam1*, *Cdh5*), epithelial (e.g. *Epcam*), or immune (e.g. *Ptprc*) marker genes.

To further validate the presence Acta1+ cells, we reanalyzed bulk RNA-Seq data from an independent study on hyperoxia-exposed P10 murine lungs. *Acta1* was markedly increased in hyperoxia (n = 6), compared to normoxia, (n = 5) P10 mice. *Tubb3*, *Aqp3*, and *Lgals3* expression was also increased by hyperoxia^59^ (Fig. 6D). In-situ hybridization imaging of lungs from a separate group of mice (n = 5 for each group) confirmed an increased density of *Acta1*+ cells in both male and female hyperoxia-exposed mice versus normal P7 mice, especially near pulmonary blood vessels (Fig. 6E). To further buttress the findings, additional in situ studies were performed on normoxic (n = 3) and hyperoxic (n = 3) P7 mice. The *Acta1*+ expressing cells, quantified as a percentage of the overall number of cells, were markedly increased in hyperoxia, compared to normoxia (Fig. 6F; P < 0.05, n = 3, student’s test). Thus, with hyperoxia a previously undetected, but transcriptionally distinct *Acta1*+ cell population emerges in the neonatal lung.

## Discussion

Mesenchymal cells are responsible for essential functions in the developing lung, including modulating the composition of the extracellular matrix, providing contractility to airways and vasculature, and supporting angiogenesis and alveolarization^5,8,26^. Single-cell transcriptomics has been recently applied by several groups to investigate the composition of the lung mesenchyme with high granularity^22,26,29,50^. Despite similar experimental approaches across studies, there have been substantial differences in the computational analyses and subsequent biological interpretations. This study sought to comprehensively define cellular identity of the perinatal lung mesenchyme in physiologic and pathophysiologic lung development.

The transcriptional identity of several mesenchymal cell subtypes was clarified. A transcriptomic signature was identified for vascular smooth muscle cells that distinguishes the population from airway smooth muscle, myofibroblasts, and pericytes^19,20,22,29^.Consistent with a prior report alveolar and adventitial fibroblasts were noted^20^ while lipofibroblasts were not identified^26,29,50^. Myofibroblasts and airway smooth muscle cells were present and transcriptionally distinct in early postnatal development. Airway smooth muscle cells, but not myofibroblasts were present at P21, suggesting that in the absence of injury ductal myofibroblasts may not persist through adulthood^50^. Intriguingly, the identification of an embryonic mesenchymal cell population expressing *Crh*, which encodes a hormone that controls systemic glucocorticoid levels via the hypothalamus—pituitary gland—adrenal gland axis, was identified computationally and in situ. These observations support prior reports and point to a novel role for the cell in promoting perinatal lung epithelial cell maturation^26,50^. Lung development proceeds normally in Crh-deficient mice through E17.5, but septal thinning and airspace formation is subsequently insufficient and newborn pups die within 24 h^60^. The significance of Crh expressing lung myofibroblast precursors remains unknown. However, a paradigm wherein cells in the lung call for systemic cortisol release to promote surfactant release immediately prior to parturition merits further study. Interrogation of the function and physiologic role with cell type-specific gain- and loss-of-Crh expression will provide meaningful insight.

While the effects of hyperoxia on the transcriptomes of lung cells have been described in previous publications^17,18^, this study focused specifically on the effects on hyperoxia on mesenchymal cells. The dramatic loss of proliferating pericytes and the subsequent alteration of cell–cell communication networks are suggestive of a potentially underappreciated role for pulmonary pericytes in protecting against neonatal lung injury^61^. While the specific functions of lung pericytes are not fully understood, pericyte deficiency can disrupt the blood–brain barrier and contributes to the capillary instability, vascular leak and macular edema of diabetic retinopathy^62,63^. Ongoing efforts by our groups are aimed at clarifying the physiologic implications of partial pericyte loss on the altered tissue structure and pathologic vascular remodeling observed during lung recovery after hyperoxia. Given that this murine model recapitulates many features of the human disease, elucidation of novel functions for understudied cell types might lead to new strategies to improve clinical care for prematurely born infants with bronchopulmonary dysplasia.

The emergence of *Acta1*-expressing cells in multiple hyperoxia-exposed mice, seen by single cell and bulk transcriptomics and confirmed via in-situ hybridization, raises questions about developmental ontogeny and putative pathophysiologic function. While much remains to be discovered about these cells, in a previous study cardiomyocyte-associated genes were differentially expressed in a bulk transcriptomic of whole lung after hyperoxia exposure^64^. Intriguingly, cardiomyocytes of the pulmonary vein, which have been associated with asthma^65^, express *Actc1* and partially *Acta1* but not *Tubb3* and *Aqp3*^23^, suggesting that *Acta1*-expressing cells might be functionally related and possibly developmentally linked to cardiomyocytes. Given the large variability in severity seen in both hyperoxia-exposed mice and infants with bronchopulmonary dysplasia—only one of our two mice sequenced via single cell RNA-Seq contained *Acta1*+ cells, it is tempting to speculate that these cells might be a stochastic response of the body restricted to the most severe forms of disease. Future experiments using transgenic mice for *Acta1*, *Tubb3*, or other marker genes specific for these cells are planned to test this hypothesis. Though prior transcriptomic studies of the neonatal lung have employed hyperoxia as a lung injury model^18^, this report is the first to identify *Acta1*+ cells in the lung. The study design likely accounts for the discovery of the *Acta1*+ cells as the cell digestion protocol was carefully optimized^11,66^. Moreover, the read depth per cell was significantly greater than reported in prior studies (Fig. S1). Each of these factors likely contributed to our ability to identify previously undetected, relatively low abundance cells.

Overall, this study constitutes a coherent cellular portrait of the perinatal lung mesenchyme that aims to overcome discrepant biologic interpretations in the existing literature and deeply characterize the cellular and transcriptional changes that accompany neonatal hyperoxia exposure, a widely used model for bronchopulmonary dysplasia. The combination of de novo high-quality data collection, integration with and comparative secondary analysis of extant data sets, and validation via in-situ hybridization provides a blueprint that could be used to systematically improve our understanding of the mesenchymal compartment across organs and organisms.

## Materials and methods

### Mouse lung cell isolation

C57BL/6 mice were obtained from Charles River Laboratories. For studies using E18.5, P1, and P7 murine lungs, pregnant dams were purchased, and pups aged prior to lung isolation. At E18.5, the dam was asphyxiated with CO2 and pups extracted. At P1, P7, and P21 pups were euthanized with euthanasia solution (Vedco Inc). For hyperoxia experiments, pups were exposed to 80% atmosphere for 7 days as previously described^17^. Genetic sex of mice at developmental stages E18.5, P1 and P7 was determined by performing PCR amplification of the Y chromosome gene Sry. P1 and P7 mice were sexed through identification of a pigment spot on the scrotum of male mice^67^. For all timepoints, female and male mice were randomly selected for the studies. For all timepoints, except E18.5, the pulmonary circulation was perfused with ice cold heparin in 1× PBS until the circulation was cleared of blood. Lungs were minced and digested with Liberase (Sigma Aldrich) in RPMI for 15 (E18.5, P1, and P7) or 30 (P21) minutes at 37 C, 200 rpm. Lungs were manually triturated and 5% fetal bovine serum (FBS) in 1× PBS was used to quench liberase solution. Red blood cells were lysed with 1× RBC lysis buffer (Invitrogen) as indicated by the manufacturer and total lung cells counted on Biorad cell counter (BioRad). Protocols were prospectively approved by the Institutional Animal Care and Use Committee at Stanford (APLAC #19087). All murine studies adhered to American Physiological Society and the United States National Institutes of Health guidelines for humane use of animals for research. Moreover, the present study is reported in accordance with the ARRIVE guidelines.

### Immunostaining and fluorescence-activated cell sorting (FACS) of single cells

Lungs were plated at 1 × 10^6^ cells per well and stained with Fc block (CD16/32, 1:100, Tonbo Biosciences) for 30 min on ice. Cells were surface stained with the endothelial marker CD31 (1:100, eBioscience/ThermoFisher), epithelial marker Epcam (1:100, eBioscience/ThermoFisher), and immune marker CD45 (1:100, eBioscience/ThermoFisher) for 30 min on ice. The live/dead dye, Sytox Blue (Invitrogen), was added to cells and incubated for 3 min prior to sorting into 384-well plates (Bio-Rad Laboratories, Inc) prefilled with lysis buffer using the Sony LE-SH800 cell sorter (Sony Biotechnology Inc), a 100 μm sorting chip (Catalog number: LE-C3110) and ultra-purity mode. Single color controls were used to perform fluorescence compensation and generate sorting gates. 384-well plates containing single cells were spun down, immediately placed on dry ice and stored at − 80 ºC.

### cDNA library generation

RNA from sorted cells was reverse transcribed and amplified using an adaptation of the Smart-Seq2 protocol for 384-well plates^11^. Concentration of cDNA was quantified using Quant-it Picogreen (Life Technologies/Thermo Fisher) to ensure adequate cDNA amplification and cDNA was normalized to 0.4 ng/μL. Tagmentation and barcoding of cDNA was prepared using in-house Tn5 transposase and custom, double barcoded indices^20^. Library fragment concentration and purity were quantified by Agilent bioanalyzer. Libraries were pooled and sequenced on Illumina NovaSeq 6000 with 2 × 100 base kits and at a depth of around 1 million read pairs per cell.

### scRNA-Seq analysis

Sequencing reads were mapped against the mouse genome (GRCm38) using STAR aligner (https://github.com/alexdobin/STAR)^68^ and gene expression was quantified using HTSeq (https://github.com/simon-anders/htseq)^69^. To coordinate mapping and counting, snakemake (https://snakemake.readthedocs.io/en/stable/) was used^70^. Gene expression count tables were converted into loom objects (https://linnarssonlab.org/loompy/) and cells with less than 50,000 uniquely mapped reads or less than 400 genes per cell were discarded. Doublets were discarded by excluding small clusters and single cells that coexpress markers for incompatible cell types. Counts for the remaining NNN cells were normalized to counts per million reads. For t-distributed stochastic embedding (t-SNE)^12^, 500 features were selected that had a high Fano factor in most mice, and the restricted count matrix was log-transformed with a pseudocount of 0.1 and projected onto the top 25 principal components using scikit-learn (https://scikit-learn.org/)^71^. Unsupervised clustering was performed using Leiden (https://github.com/vtraag/leidenalg) (C++/Python implementation)^24^. Singlet (https://github.com/iosonofabio/singlet) and custom Python3 scripts were used: the latter are available at https://github.com/iosonofabio/lung_neonatal_mesenchymal/. Pathway analysis on the differentially expressed genes was performed via Metascape^38^ on the top 100 most differentially expressed genes for each comparison: precursors versus sample-balanced joint progenies and subsequent time points within each cluster. The most enriched pathways against a permutation test are shown ordered by significance from top to bottom (negative log of the P-value). Batch-corrected KNN^25^ was used to compare our P21 data with the Smart-seq 2 data from Tabula Muris Senis^19^.

### In-situ validation using RNAscope and immunofluorescence (IF)

Embryonic and post-natal mice were euthanized as described above. Female and male mice were randomly selected from the litter, and at least two litters were used to source the lung tissue for all validation studies. E18.5 lungs were immediately placed in 10% neutral buffered formalin following dissection. P1, P7, and P21 murine lungs were perfused as described above, and P7 and P21 lungs inflated with 2% low melting agarose (LMT) in 1× PBS and placed in 10% neutral buffered formalin. Following 20 h incubation at 4 °C, fixed lungs were washed twice in 1× PBS and placed in 70% ethanol for paraffin-embedding. In situ validation of genes identified by single cell RNA-seq was performed using the RNAscope Multiplex Fluorescent v2 Assay kit (Advanced Cell Diagnostics) and according to the manufacturer’s protocol. Formalin-fixed paraffin-embedded (FFPE) lung section (5 μm) were used within a day of sectioning for optimal results. Nuclei were counterstained with DAPI (Life Technology Corp.) and extracellular matrix proteins stained with hydrazide^72^. Opal dyes (Akoya Biosciences) were used for signal amplification as directed by the manufacturer. Images were captured with Zeiss LSM 780 and Zeiss LSM 880 confocal microscopes, using 405 nm, 488 nm, 560 nm and 633 nm excitation lasers. For scanning tissue, each image frame was set as 1024 × 1024 and pinhole 1AiryUnit (AU). For providing Z-stack confocal images, the Z-stack panel was used to set z-boundary and optimal intervals, and images with maximum intensity were processed by merging Z-stacks images. For all both merged signals and split channels were collected.

### Image quantification

Images were quantified in two ways. First, for pericytes we used automatic image segmentation using cellprofiler^73^ to identify RNA molecules from RNA-Scope and the cell boundaries, and then counted the number of cells with at least 3 positive RNA molecules in different conditions. Second, for other cells that were challenging to segment (e.g. ASM/MyoF), whole-image correlations of the fluorescence channels were computed.

### In-situ validation of pericyte abundance using transgenic mice

Notch3-CreERT2-tdT mice were given tamoxifen at P0 and P3 and placed in normoxia or hyperoxia (0.80 FiO2) from P1 to P7. Lungs were harvested at P7 and inflation fixed. Deep imaging of thick lung section (150 μm) allowed us to visualize the abundance and location of tdT-labeled pericytes in the distal lung.

### Statistical analyses

Single cell omics data do not follow a normal distribution and are often difficult to parameterize altogether. Therefore, to ensure statistical soundness of all analyses, a statistical plan based on nonparametric tests was deployed throughout the study, exploiting the fact that hundreds of quasi-replicate cells were sampled. To identify differentially expressed genes within cell populations, between time points, and between normoxia and hyperoxia, nonparametric Kolmogorov–Smirnov (KS) tests on the distributions of gene expression were performed, and either the genes with the largest absolute value of the test statistic or the genes above a certain KS statistic (e.g. 0.3) with the largest average fold change were chosen. Nonparametric bootstrapping was used to evaluate the developmental arrest of hyperoxia. Correlation tests and proportion tests for cell population abundances were used in some image analyses.

### Supplementary Information

Supplementary Figures.

## Data Availability

Raw fastq files, count tables, and metadata are available on NCBI’s Gene Expression Omnibus (GEO) website: GSE172251 and GSE175842 (immune cells). An h5ad file with the processed data is available on FigShare and can be accessed and downloaded at: https://figshare.com/articles/dataset/Cell_atlas_of_the_murine_perinatal_lung/14703792. Bulk transcriptomic data were sourced from and are available in Ref.^59^.
