# Supplementary material for: Hyperoxia prevents the dynamic neonatal increases in lung mesenchymal cell diversity
Source: Sci Rep. 2024 Jan 23;14:2033. doi: 10.1038/s41598-023-50717-w (PMC10805790; doi:10.1038/s41598-023-50717-w)
Supplement: Supplementary file 1 — Supplementary Figures. [file 41598_2023_50717_MOESM1_ESM.pdf]

Supplementary text and figures for:

Lung mesenchymal cell diversity rapidly increases at birth and is profoundly altered by hyperoxia

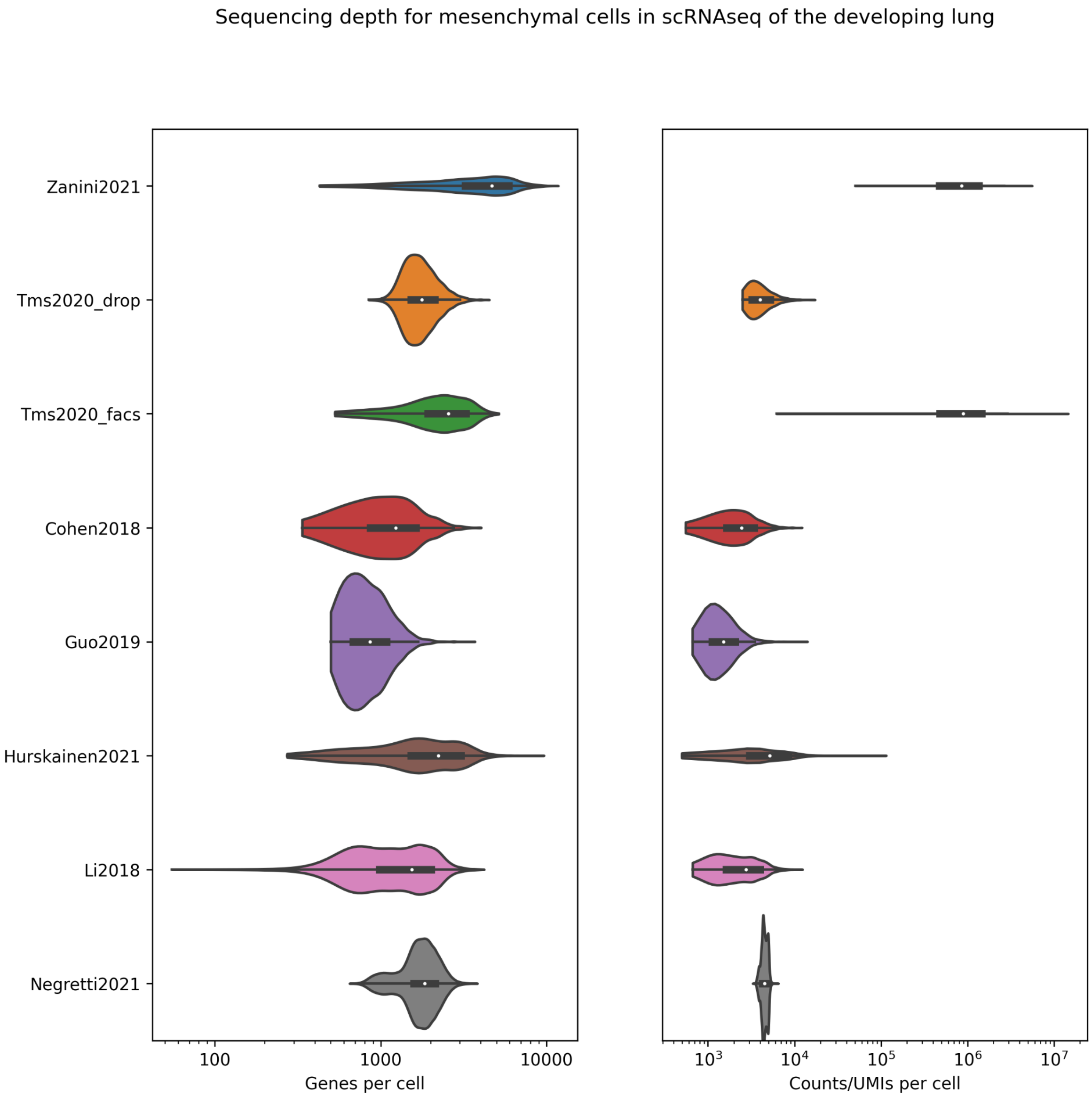

Figure S1. Violinplot with numbers of genes per cell and number of reads/UMIs per cell for mesenchymal cells from several scRNA-Seq data sets from murine lungs.

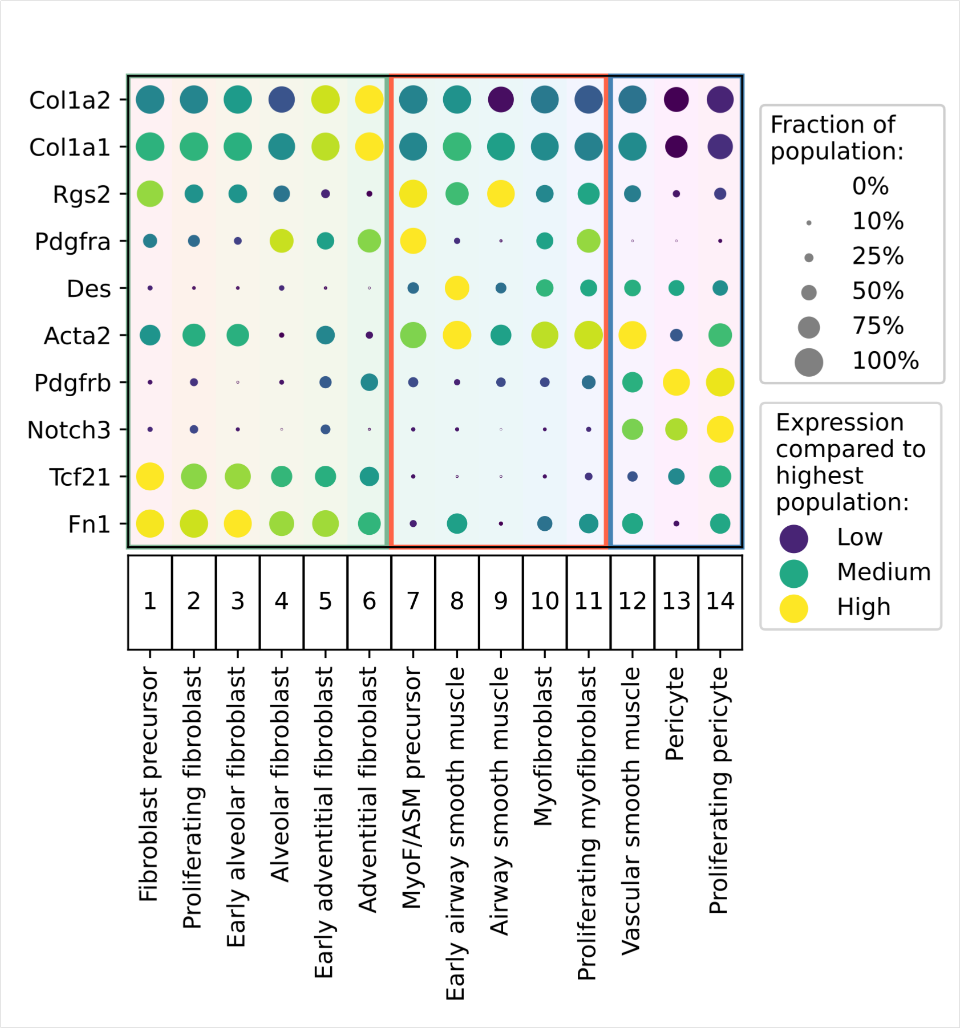

Figure S2. Dot plot of the marker genes by Guo et al. across our subtypes. These genes are the same as in ref [31], Figure 1b, limited to the markers for their mesenchymal populations.

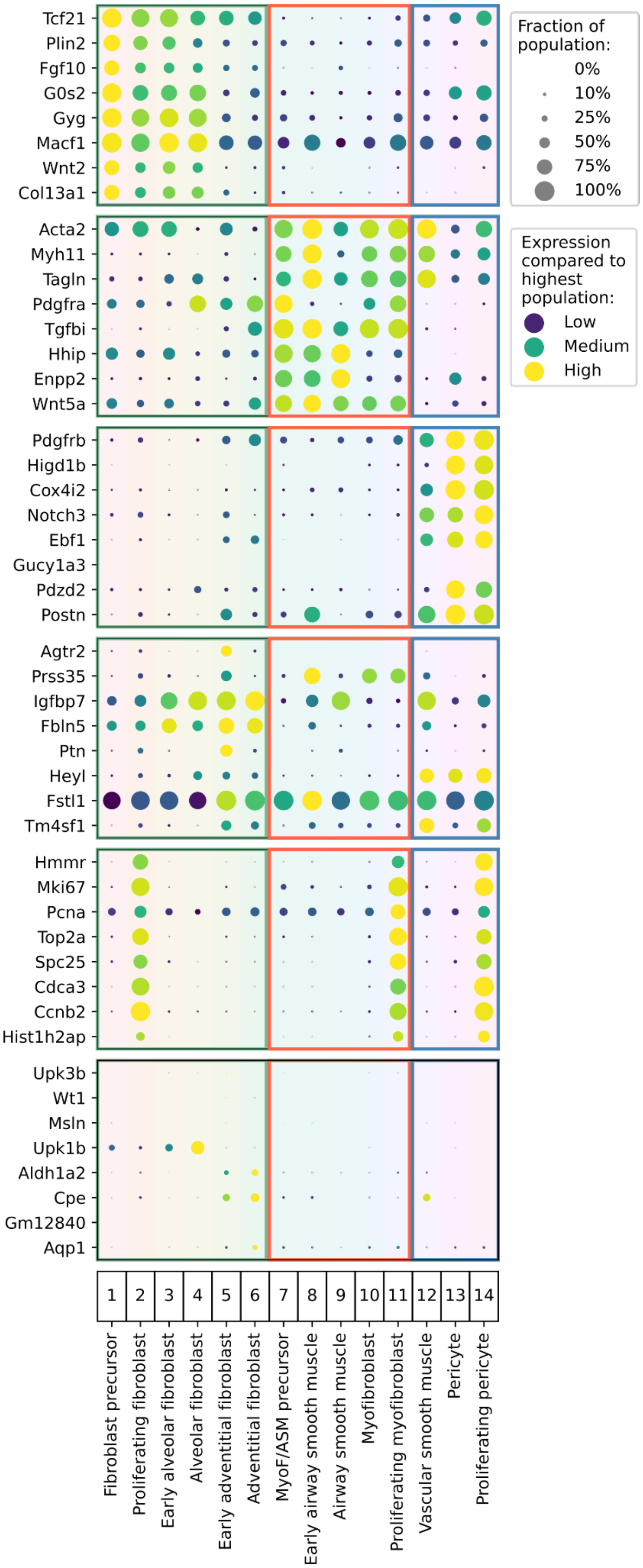

Figure S3. Dot plot of the marker genes by Liu et al. across our subtypes. These genes are the same as in ref [23], Figure 1E.

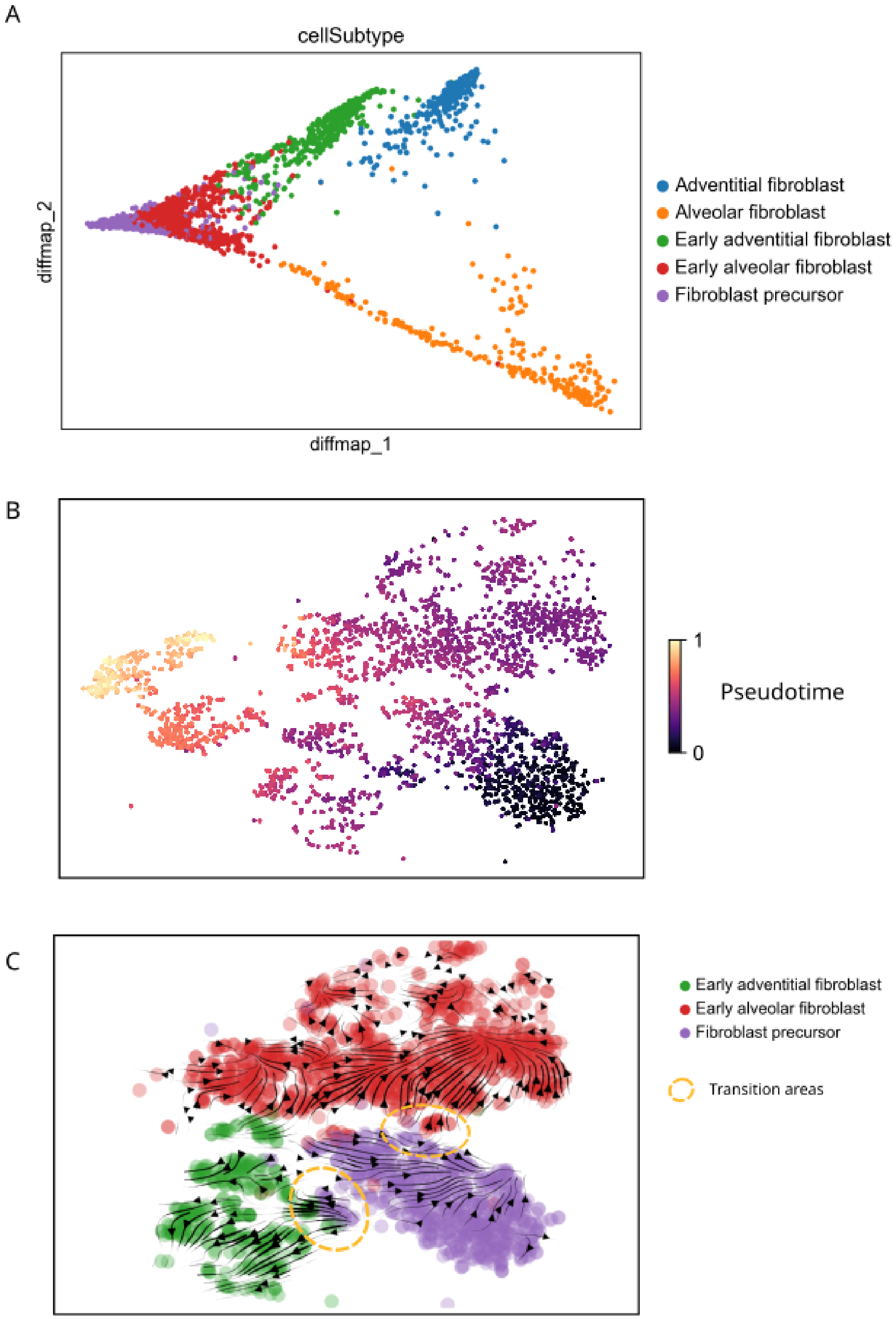

Figure S4. Trajectory analysis of fibroblast precursors and early postnatal fibroblasts. (A) Diffusion map colored by MS subtype. (B) Pseudotime analysis (Wolf et al., Genome Biology 2019) starting from fibroblast precursors. (C) RNA velocity stream analysis using scVelo Bergen *et al*. Nature Biotech 2020) with transitional areas between precursors and postnatal fibroblasts highlighted.

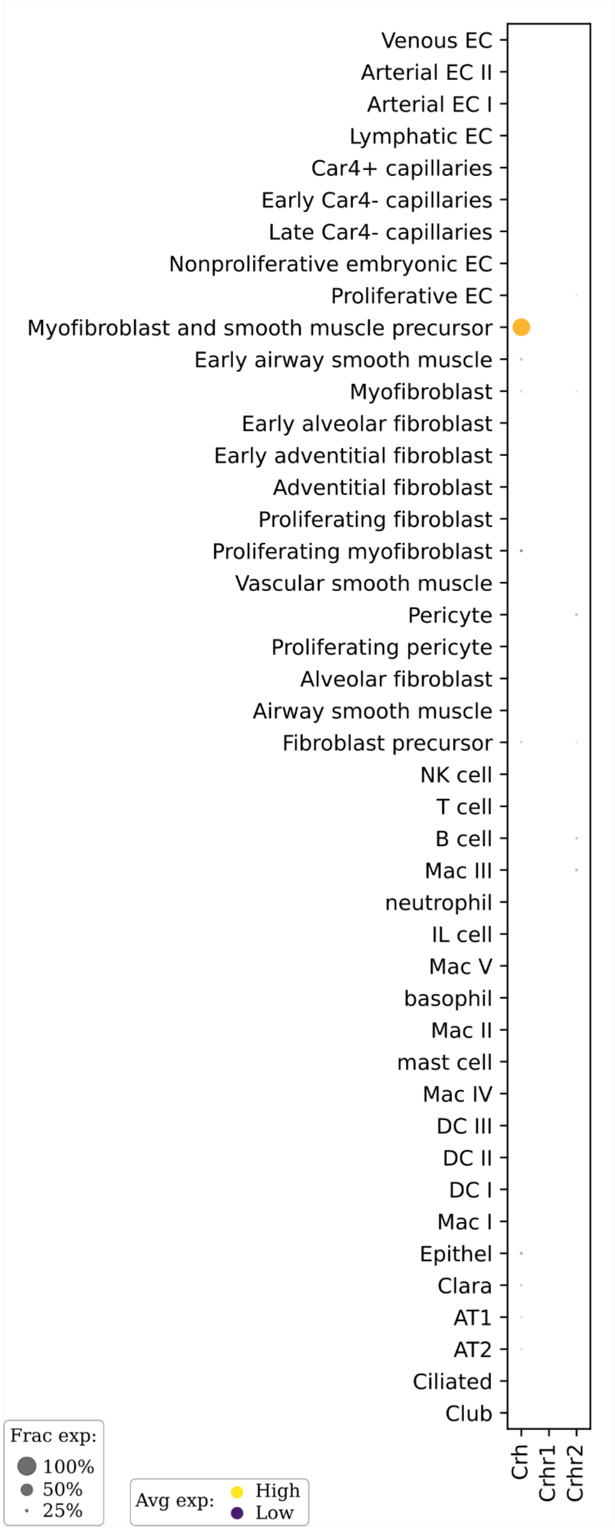

Figure S5. Dot plot of Crh and its receptors in developing murine lungs. Mesenchymal cell data from this article, endothelial cells from [18], immune cells from [11], epithelial from [44]. Neither of the receptors is expressed in the developing lung in any detected cell type.

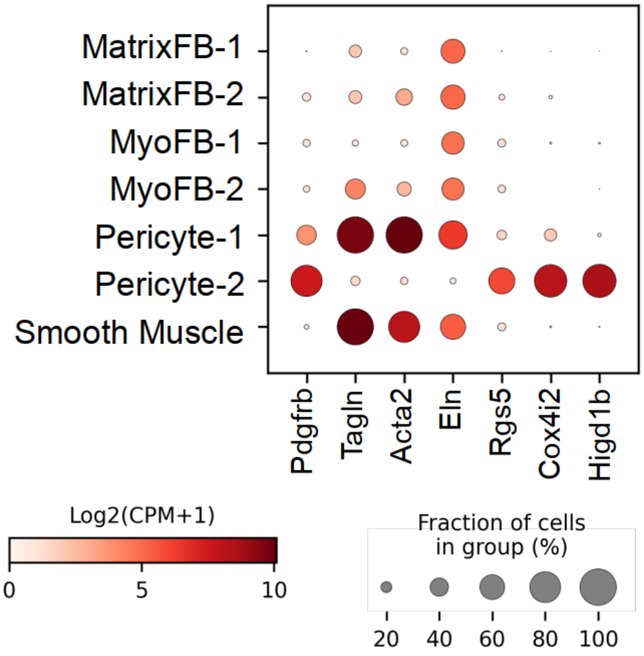

Figure S6: Dot plot for pericyte and VSM marker genes in Guo et al [31].

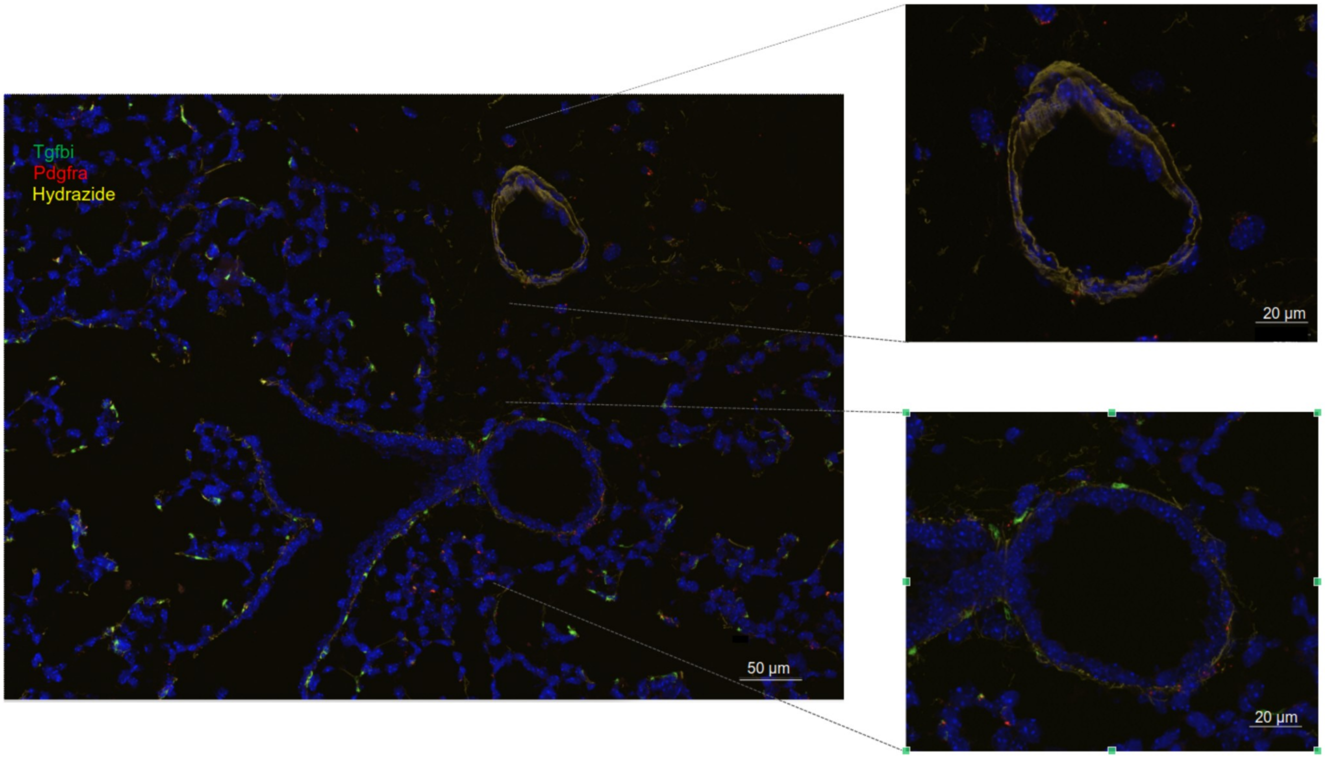

Figure S7. *In situ* hybridization of lung at P7 to detect Tgfbi (green), a marker for ASM/MyoF but not VSM, Pdgfra (red) and hydrazide (yellow). The enlarged zoom-ins show a blood vessel (top) and an airway (bottom).
